# Dental Glass Ionomer Cement for Root Perforation Management: Physicochemical Characteristics and In Vitro Cell Response

**DOI:** 10.3390/dj14050284

**Published:** 2026-05-09

**Authors:** Alexandra Popa, Radu-Vasile Radulescu, Florentina Rus, Vlad Gabriel Vasilescu, Lucian Toma Ciocan, Monica Musteanu, Marina Imre, Silviu Pituru, Ana Cernega, Alexandra Ripszky, Ecaterina Andronescu

**Affiliations:** 1 The Interdisciplinary Center for Dental Research and Development, Faculty of Dental Medicine, “Carol Davila” University of Medicine and Pharmacy, 19-21 Jean Louis Calderon Street, 020021 Bucharest, Romania; alexandra.popa@umfcd.ro (A.P.); florentina.rus-hrincu@umfcd.ro (F.R.); marina.imre@umfcd.ro (M.I.); ana.cernega@umfcd.ro (A.C.); alexandra.ripszky@umfcd.ro (A.R.); 2 Department of Biochemistry, Faculty of Dental Medicine, University of Medicine and Pharmacy Carol Davila, 37 Dionisie Lupu Street, District 2, 020021 Bucharest, Romania; radu.radulescu@umfcd.ro; 3 Discipline of Dental Prosthesis Technology, Faculty of Dentistry, “Carol Davila” University of Medicine and Pharmacy, Dionisie Lupu Street, No. 37, District 2, 020021 Bucharest, Romania; lucian.ciocan@umfcd.ro; 4 Department of Biochemistry and Molecular Biology, Faculty of Pharmacy, University Complutense of Madrid, 28040 Madrid, Spain; mmustean@ucm.es; 5 Department of Complete Denture, Faculty of Dental Medicine, University of Medicine and Pharmacy Carol Davila, 17-23 Calea Plevnei, 010221 Bucharest, Romania; 6 Department of Professional Organization and Medical Legislation-Malpractice, “Carol Davila” University of Medicine and Pharmacy, 020021 Bucharest, Romania; 7 Department of Science and Engineering of Oxide Materials and Nanomaterials, Faculty of Chemical Engineering and Biotechnologies, National Polytechnic University of Science and Technology of Bucharest, 011061 Bucharest, Romania; ecaterina.andronescu@upb.ro; 8 National Research Center for Micro and Nanomaterials, National Polytechnic University of Science and Technology of Bucharest, 060042 Bucharest, Romania; 9 Romanian Academy of Scientists, 050045 Bucharest, Romania

**Keywords:** root perforation, biocompatibility, FT-IR, glass ionomer, XRD

## Abstract

**Background/Objectives:** Root perforation treatment is essential for restoring the tightness of the root system, preventing periradicular inflammation and tooth loss. The present study aimed to evaluate the biocompatibility of Ketac™ Molar EasyMix as well as conduct a thorough morphological and structural characterization of the material, considering its potential use in managing root perforations. **Methods:** Morpho-structural characterization was performed by scanning electron microscopy (SEM), energy dispersive X-ray spectroscopy (EDX), Fourier-transform infrared spectroscopy (FT–IR), and X-ray diffraction (XRD). Biocompatibility tests were performed on osteosarcoma cell line (ATCC—G 292 CRL-1423), monitoring metabolic activity and cell viability (MTT, *n* = 3), as well as the inflammatory response (nitric oxide—NO, *n* = 6), after 24 and 48 h of incubation. The control group consisted of cells unexposed to the material. **Results:** Microstructurally, the material exhibits a heterogeneous structure, along with pores and cracks. The specific bonds of the material, including both organic acid (COO^−^, O-H) and the glass components (Si-O-Al, Ca-O, C-F), were identified by FT-IR, while the crystalline phase composed of calcium fluorolanthanate was determined by XRD. Moreover, in vitro metabolic activity and viability test (MTT) showed a decrease of ~28% (*p* = 0.029) and ~30% (*p* = 0.150) after 24 and 48 h for samples incubated with Ketac™ Molar EasyMix. The statistically significantly increased levels of NO (*p* = 0.002, *p* = 0.004) suggest that the cells are trying to adapt to the environment that they have been exposed to. **Conclusions:** Within the limitations of the present study, under the tested conditions, our results suggest that Ketac™ Molar EasyMix maintained cell viability close to the 70% threshold defined by ISO 10993-5:2009, indicating a borderline biological response, a feature that may be influenced by the composition and behavior of the material.

## 1. Introduction

Root perforation remains one of the most complex and clinically challenging complications encountered in modern endodontics. It is defined as abnormal communication between the endodontic space and the surrounding periodontal tissues, leading to a loss of root wall integrity and providing a direct pathway for inflammatory irritation and microbial contamination [1,2]. Under physiological conditions, root dentin and cementum act as a barrier that separates the pulp–root canal system from the periodontium. When this barrier is disrupted, the perforation site may rapidly become colonized, triggering an inflammatory response characterized by cell infiltration, breakdown of periodontal attachment, and alveolar bone loss, with potential progression towards tooth loss in unfavorable cases [3,4]. Perforations have been consistently recognized as a key factor in endodontic failure; evidence indicates that up to ~10% of root canal treatment failures may be associated with perforation events, underscoring their significant clinical importance [1,5].

Perforations can be classified according to etiology, anatomic location, defect size, and the extent and duration of microbial contamination, factors that also impact prognosis and treatment choices [6]. Etiologically, perforations can be iatrogenic, occurring during procedures like access cavity preparation, canal negotiation and shaping, or post-space preparation, particularly in teeth with complex root canal systems, calcifications, or compromised canal patency [7,8]. Additionally, perforations may result from pathological conditions such as deep caries or internal/external resorption, which gradually weaken the root’s structural integrity [9]. Clinically, perforations may manifest as persistent bleeding, sudden pain during probing or instrumentation, unexpected communication during treatment, or sinus tract formation. Diagnosis and localization often involve radiographs and three-dimensional imaging, especially for defects that are difficult to visualize directly [1,10].

From a therapeutic standpoint, the management of perforations aims to restore root continuity and prevent (or limit) bacterial contamination of the periodontium—goals that align with the fundamental objective of endodontic therapy: establishing a durable seal that promotes healing [11]. Classical prognostic discussions emphasize that outcomes depend strongly on the time to repair, defect size, and defect location, as delayed sealing and cervical/crestal defects generally carry a higher risk of persistent periodontal breakdown compared with small apical defects sealed promptly [2,12]. Consequently, the repair material must exhibit biocompatible properties, provide effective adhesion and sealing against dentine, remain dimensionally stable, and perform reliably in the moist environment characteristic of periodontal and surgical fields [13].

In perforations located in the apical third, or when orthograde access is limited or compromised, surgical endodontic procedures may be required, with retrograde obturation (root-end filling) as a central step in modern endodontic surgery [14]. In a standard microsurgical workflow, the procedure involves root-end resection (apicoectomy), followed by retrograde cavity preparation—often performed with ultrasonic tips to optimize access and cavity geometry—and placement of a root-end filling material [15,16]. The retrograde filling must prevent bacterial migration from the canal space into periapical tissues and should create an environment conducive to periapical healing. In this context, treatment success is closely linked not only to surgical execution but also to material properties—chemical and physical stability, sealing performance, and biological behavior under direct contact with periodontal tissues [14,17].

Over the years, various materials have been used for perforation repair and retrograde obturation. In recent decades, calcium silicate-based “bioceramic” materials—such as mineral trioxide aggregate (MTA) and similar hydraulic cements—have become popular in endodontics due to their excellent sealing ability and bioactivity [18,19]. These hydrated calcium silicate cements can release calcium ions and generate calcium hydroxide. In phosphate-containing environments, these reactions may lead to the formation of calcium phosphate deposits that mature into hydroxyapatite-like structures, often considered to enhance interfacial sealing and biological integration [20]. Consequently, the current clinical literature frequently regards these bioceramics as among the best options for sealing perforations and root-end cavities [2,18].

Despite the broad diffusion of bioceramics, glass ionomer cements (GICs) have played—and continue to play—an important role in restorative dentistry and remain relevant for selected applications involving root defects, particularly because of their unique adhesion chemistry and ion release profile [21]. The first glass ionomer cement was introduced by Wilson and Kent (1972) as a dental cement capable of chemically bonding to mineralized tissues [22]. Conventional GICs typically consist of a fluoroaluminosilicate glass powder and an aqueous polyalkenoic acid solution (commonly polyacrylic acid or copolymers/copolymers), and they set through an acid–base reaction [23]. During setting and maturation, glass degradation leads to the release of ions (e.g., Ca^2+^, Al^3+^, and F^−^), formation of a cross-linked matrix, and development of an ion-exchange interaction at the tooth interface that supports chemical adhesion; importantly, maturation processes continue beyond the initial hardening stage and can influence longer-term properties [24].

A further clinically relevant feature is the capacity of GICs to release fluoride over prolonged periods, a phenomenon extensively documented and associated—depending on context—with anti-caries and potential antimicrobial/remineralizing effects in restorative settings [25]. Fluoride release is not uniform across products and is affected by formulation and environmental conditions; consequently, individual materials may show distinct ion release kinetics and biological interactions [23,26]. For instance, controlled in vitro comparisons have reported that Ketac™ Molar Easymix exhibits among the highest fluoride release values within tested GIC groups, while antibacterial effects may not necessarily scale proportionally with fluoride release, emphasizing the need for material-specific evaluation rather than generalization across the GIC class [27].

In endodontics, the early use of GICs for perforation management is sometimes limited by concerns related to their mechanical properties, solubility during initial setting, and water sensitivity compared to hydraulic bioceramics [28]. However, the literature also shows that, when used under handling conditions and appropriate indications, GICs can provide effective sealing and favorable tissue responses. Additionally, experimental studies have investigated GICs as retrograde filling materials and assessed tissue reactions in vivo [29,30]. A potential advantage in surgical practice is that their chemical adhesion to mineralized tissues may aid handling even with imperfect isolation, which is a common issue during periapical surgery since moisture control is more challenging than in coronal restorations [28,30].

Beyond adhesion and fluoride release, there is ongoing interest in whether certain GIC formulations can exhibit bioactive behavior at tissue interfaces. Apatite-like layer formation has been reported in simulated body fluid for specific glass ionomer-related materials (notably resin-modified formulations), suggesting that ion release and interfacial reactions may support mineral deposition under certain conditions [23,30,31]. Additionally, modifications of glass ionomer systems with bioactive glass have been explored to increase bioactivity and influence mineral interactions, further reinforcing the concept that compositional and formulation differences can meaningfully change biological behavior [32,33].

At the same time, the biocompatibility of GICs cannot be assumed uniformly across products, because cytotoxicity profiles depend on leachable components, maturation stage, and experimental conditions. In vitro investigations have compared multiple commercial GICs using cellular models relevant to oral tissues, including gingival fibroblasts and osteoblast-lineage cells, and reported material-dependent variations in cell viability and response [34,35]. Critically, osteoblast and osteosarcoma-related data remain relatively limited for specific formulations used in clinical practice, and the available evidence supports the need for product-specific evaluation—particularly for materials proposed for scenarios involving direct contact with bone-associated tissues [35]. Cytotoxic effects on osteoblast-lineage cells may affect cytokine signaling and the RANKL/OPG ratio, promoting osteoclastogenesis and bone resorption [35].

Root perforations therefore represent a complex pathology with major implications for dental prognosis, where the choice of repair material may decisively influence treatment outcomes [1,2,8,9]. Considering the sustained clinical use of GICs and the material-specific variability in physicochemical behavior and biological interactions, further evaluation of contemporary high-viscosity GIC formulations is justified, especially in contexts such as root defects and retrograde obturation where contact with periodontal/periapical tissues is expected [21,23,25]. The correlation of physicochemical features with the cellular response may provide a more comprehensive understanding of biocompatibility and of the potential of this material to support tissue integration in clinical scenarios involving root perforations and retrograde obturation.

Based on these considerations, the present study aimed to investigate the impact of Ketac™ Molar glass ionomer cement on osteosarcoma-lineage cells by assessing functional outcomes related to metabolic activity/cell viability and inflammatory-related response (nitric oxide release), alongside detailed structural and morphological characterization of the material. The null hypothesis (H_O_) of this study was that Ketac™ Molar EasyMix would not induce statistically significant changes in cell viability or nitric oxide production compared to unexposed control cells, while maintaining ≥70% cell viability in G292 cells according to ISO 10993-5:2009, and it exhibits physicochemical features compatible with use in root perforations and retrograde fillings.

## 2. Materials and Methods

The examined material was Ketac™ Molar EasyMix ART (3M ESPE/Solventum, St. Paul, MN, USA; produced in Germany), a classic glass ionomer cement. Its composition, listed in Table 1, is derived from the manufacturer’s data sheet.

### 2.1. Sample Preparation

Twenty-three circular samples, 7 mm in diameter and 2 mm thick, were made using polyvinyl chloride molds, following the manufacturer’s instructions (component ratio—4.5 parts powder (one level spoonful): one part liquid (one drop)). The mixing of the two components was performed on a glass plate using a metal spatula until a homogeneous mass was obtained. They were allowed to set for 5 min at 37 °C with 5% CO_2_ and were subsequently disinfected by UV exposure for 10 min on each side to reduce the risk of bacterial colonization of the cell cultures. After this time interval, the samples were immediately tested, as follows: 6 disks were used for the MTT assay (3 disks × 2 incubation times), 12 disks were used for NO (6 disks × 2 incubation times), and one disk each was allocated for the SEM, EDX, FT-IR, and XRD investigations. One more disk was allocated for SEM analysis at 14 days. Until day 14, it was stored in the incubator at 37 °C with 5% CO_2_ to be subsequently examined.

### 2.2. Morphological and Structural Characterization

#### 2.2.1. Scanning Electron Microscopy (SEM) and Energy Dispersive X-Ray Spectrum (EDX)

The morphological and dimensional features were examined using a QUANTA INSPECT F50 microscope (Thermo Fisher, Eindhoven, the Netherlands). This microscope is equipped with an energy dispersive X-ray spectrometer (Gatan, Inc., CA, USA), enabling elemental analysis with a 133 eV resolution at the Mn Kα line. It also features a field-emission electron gun (FEG), achieving a resolution of 1.2 nm for detailed nanoscale imaging.

#### 2.2.2. Fourier-Transform Infrared Spectroscopy (FT–IR)

The vibrations of the functional groups were analyzed using a Thermo Nicolet 6700 FT-IR spectrometer (Thermo Fisher Scientific, Waltham, MA, USA) with a ZnSe crystal. For the FT-IR analysis, the material was mixed, allowed to set, and then ground into a fine powder for measurement. These measurements were performed at room temperature, with 32 scans per sample to ensure accuracy and reproducibility. Spectral data were collected at a resolution of 4 cm^−1^, over a 4000–400 cm^−1^ range. The instrument was controlled by using Omnic software (Thermo Nicolet, v.8.2—Thermo Fisher Scientific, Waltham, MA, USA), connected to a processing system and database for precise IR spectrum interpretation. 

#### 2.2.3. X-Ray Diffraction (XRD)

The crystalline phases were determined through XRD analysis via a PANalytical Empyrean diffractometer (Malvern PANalytical, Bruno, The Netherlands). This instrument was provided with a hybrid monochromator (2xGe 220) on the incident beam, ensuring accurate control of X-radiation. On the diffracted side, a parallel plate collimator mounted on a 3D PIXcel detector was used, enhancing measurement accuracy. Experiments were conducted at room temperature with an incidence angle of 0.5°, over a Bragg 2θ angle from 10° to 80°. Each acquisition was performed with a collection time of 255 s and a step size of 0.01414° for detailed results. The Cu Kα radiation source (λ = 1.5406 Å) operated at 40 mA and 45 kV.

### 2.3. Metabolic Activity, Viability and Cytotoxicity

#### 2.3.1. Cell Culture

Human osteosarcoma cells (G 292 CRL-1423), from ATCC (10801 University Boulevard, Manassas, VA, USA), were cultured in 75 cm^2^ flasks using DMEM/F12 (Dulbecco’s Modified Eagle Medium, Sigma-Aldrich^®^ Solutions, Merck KGaA, Darmstadt, Germany) enhanced with 1% antibiotic and antifungal and 10% fetal bovine serum. The flasks were kept in a humidified incubator with 5% CO_2_ at 37 °C, and the medium was replaced every 3 days. 

Cells (G292) were distributed at a seeding density of 10^4^ per well in a 24-well plate and incubated overnight to allow attachment. Subsequently, the cells were incubated with the dental material for 24 and 48 h at 37 °C with 5% CO_2_. The control group consisted of cells grown in a DMEM/F12 medium without any material. The cells were directly exposed to the material samples.

The G292 human osteosarcoma cell line maintains essential functional characteristics of osteoblast-lineage cells, such as expressing osteoblast markers and displaying metabolic activities relevant to bone tissue responses. Additionally, previous research has utilized osteosarcoma-lineage and osteoblast-related cell models to evaluate the biocompatibility of glass ionomer cements [31,35]. Procedures like root perforation management and retrograde obturation involve the direct contact of the repair material with periapical tissues, where responses from osteoblast-lineage cells influence local inflammation, adaptation, and healing. Therefore, assessing the biological effects of Ketac™ Molar EasyMix on an osteoblast-related model is scientifically justified and directly applicable to the clinical conditions [31,35].

#### 2.3.2. Metabolic Activity and Viability Assay

The MTT solution (3-(4,5-dimethylthiazol-2-yl)-2,5-diphenyltetrazolium bromide—MTT; Sigma-Aldrich, Darmstadt, Germany) was used to assess metabolic activity and viability, at a concentration of 1 mg/mL. The principle is that only metabolically active cells reduce MTT to formazan, which appears purple. After two hours, the purple formazan crystals were solubilized in isopropanol and were quantified spectrophotometrically at 595 nm using a FLUOstar^®^ Omega multi-mode microplate reader (BMG LABTECH, Ortenberg, Germany) [38,39].

#### 2.3.3. Level of Nitric Oxide (NO)

Nitric Oxide (NO) levels were measured using the Nitric Oxide Kit (Abnova, Taipei City, Taiwan) REF: KA1641, to detect nitrites and nitrates in the culture medium. Nitrates are enzymatically reduced to nitrites by nitrate reductase in the presence of NADPH. The resulting nitrites then react with sulfanilamide and N-(1-naphthyl)-ethylenediamine dihydrochloride to form a red-violet chromophore. Consequently, the nitrite concentration serves as an indicator of nitric oxide levels [39,40]. The reaction took place per well after adding the sample:reagent in a ratio of 1:2 and incubated for 10 min at 60 °C. Subsequently, the results were quantified spectrophotometrically at 540 nm using a FLUOstar^®^ Omega multi-mode microplate reader (BMG LABTECH, Ortenberg, Germany).

### 2.4. Statistics

The results were expressed relative to the control wells (100%) and were graphically represented. The graphical illustrations were generated using the arithmetic mean values for each experimental condition, and variability was expressed as mean ± standard deviation (SD). Data processing was performed using Microsoft Office Excel (Microsoft Corporation, One Microsoft Way, Redmond, WA, USA), while statistical analysis was carried out using IBM SPSS Statistics version 26.0 (IBM, New York, NY, USA). The distribution of quantitative variables was assessed using the Shapiro–Wilk test, indicating a normal distribution for all analyzed groups at both time points (24 and 48 h). The homogeneity of variances was evaluated using Levene’s test. Statistical analyses were performed separately for each parameter (MTT and NO) and for each time point (24 and 48 h), comparing the experimental groups (cells exposed to Ketac™ Molar EasyMix) with the control group (unexposed cells). Differences between groups were analyzed using one-way analysis of variance (One-Way ANOVA), and the level of statistical significance was set at α = 0.05.

## 3. Results

### 3.1. Surface Characterization and Chemical Composition

**Figure 1:**
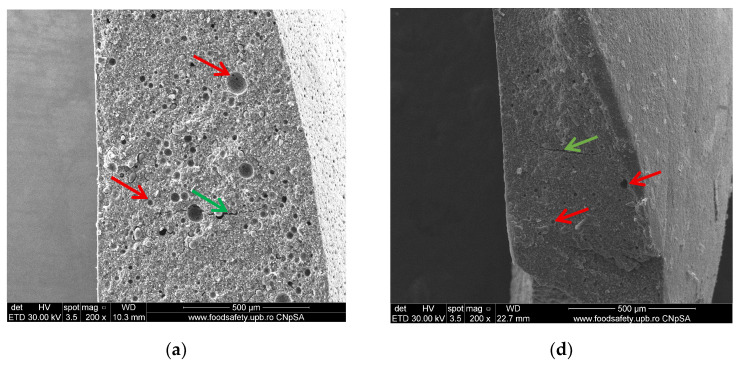

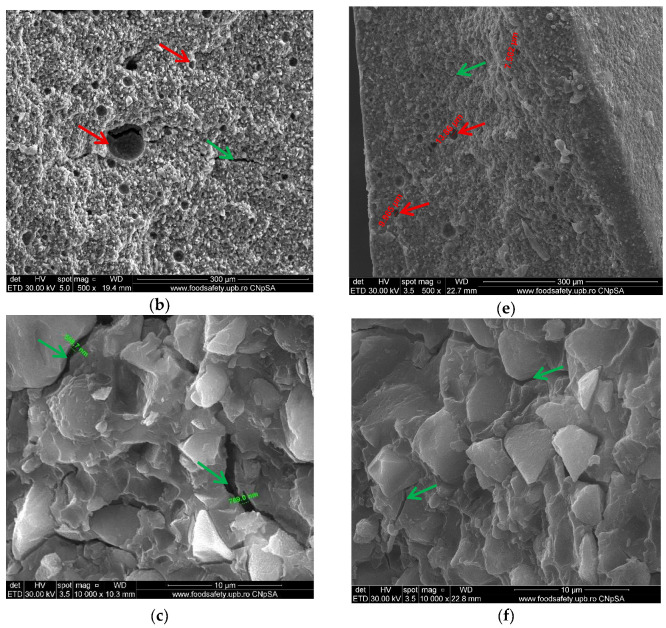
SEM micrography of Ketac™ Molar EasyMix recorded immediately after setting at magnifications of ×200 (**a**), ×500 (**b**), and ×10,000 (**c**) and after 14 days at magnifications of ×200 (**d**), ×500 (**e**), and ×10,000 (**f**). The red arrows mark the pores, and the green arrows indicate the cracks.

SEM images at 200×, 500×, and 10,000× magnification of the immediately set material reveal a heterogeneous microstructure, characterized by inorganic glass particles of variable sizes (114.9 nm–5.419 μm) that are unevenly distributed, embedded within a porous matrix represented by polyacrylic acid. A series of particles appears detached from the polymer matrix, indicating a low adhesion between the two phases or an inadequate ratio between them. This aspect can significantly affect both the mechanical strength and the sealing capability of the material. Fine fracture lines (598.7–769.0 nm), pores, and cavities can be detected outside and inside the material as well, and the pores are likely generated from the mixing process (Figure 1a–c). Cracks may also be developed due to a lack of compatibility between the way polyacrylic acid and glass particles react or from their ratios.

Porosities with dimensions between 7.562 μm and 13.86 μm, as well as fine fracture lines, are maintained even 14 days after the material sets (Figure 1d–f), without any additional structural changes being highlighted in relation to the structure observed immediately after setting.

**Figure 2:**
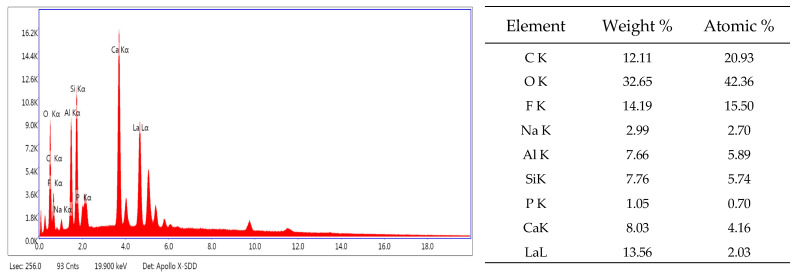
Energy dispersive X-ray spectrum related to the microarea of the Ketac™ Molar EasyMix.

The EDX (Figure 2) mainly reveals the chemical elements characteristic of glass-based material, including silicate (Si), aluminum (Al), calcium (Ca), fluorine (F), sodium (Na), and lanthanum (La).

**Figure 3:**
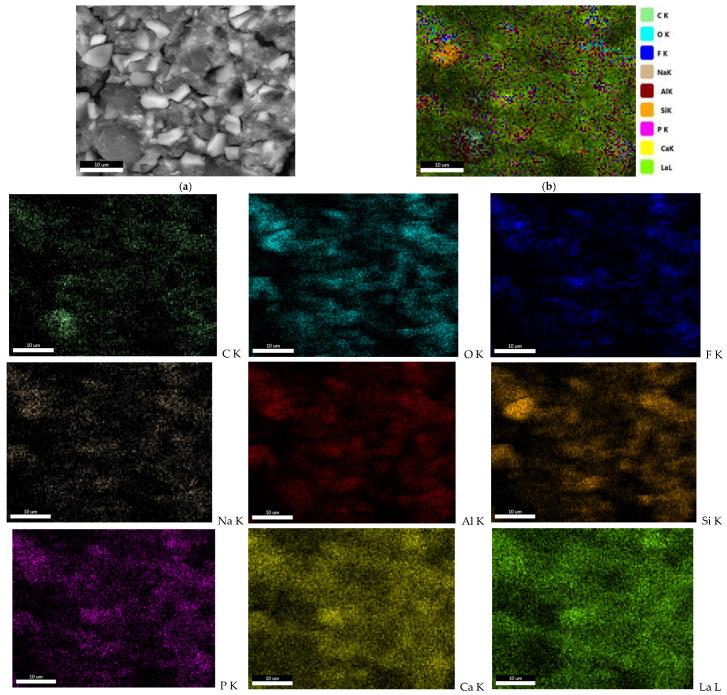
Elemental mapping recorded for Ketac™ Molar EasyMix. The backscattered electron images (**a**) and surface distribution images (**b**) of the relative intensity of X-ray radiation specific to the significant elements detected, C Kα, O kα, F Kα, Na Kα, Al Kα, Si Kα, Ca Kα, and La Lα, on the microarea of the Ketac™ Molar EasyMix sample.

Elemental mapping analysis reveals a differentiated spatial distribution of the elements: calcium and lanthanum are fairly uniformly distributed across the analyzed surface, whereas C (carbon), O (oxygen), Na, F, Al, Si, and P (phosphorus) exhibit localized enrichment in well-defined areas (Figure 3). These compositional variations correlate with the heterogeneous microstructure observed by SEM, indicating that the observed heterogeneity results from a phase-specific elemental distribution rather than a globally uneven mixture of elements. Such behavior suggests the presence of chemically distinct domains within the glass matrix, consistent with the complex microstructural features revealed by SEM.

### 3.2. FT-IR

**Figure 4:**
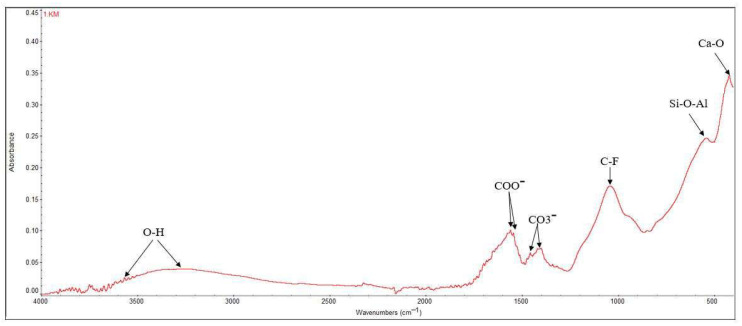
FT-IR spectra recorded for Ketac™ Molar EasyMix.

The FT-IR spectroscopy reveals the vibrations of the functional groups specific to the material made of polyacrylic acid and glass particles. Figure 4 displays the FT-IR spectrum for Ketac Molar.

Several wavelengths have been identified (Figure 4), the maximum intensity bands appearing at the Ca-O bond, 408 cm^−1^, and are mainly found at wavelengths between 300 and 600 cm^−1^. Si-O-Al bonds can be observed at 536 cm^−1^, while C-F bonds appear at wavelengths of 1039 cm^−1^. The specific bond of the carbonate ion (CO^3−^) is detected at 1456 cm^−1^, the carboxylate ion (COO^−^) at 1558 cm^−1^, and the O-H bonds, most likely from polyacrylic acid, at wavelengths of approximately 3565 and 3273 cm^−1^. The FT-IR analysis highlights both the bonds of the organic component, namely polyacrylic acid, and the bonds of the glasses in the composition of the analyzed material [41,42].

### 3.3. XRD Analysis

**Figure 5:**
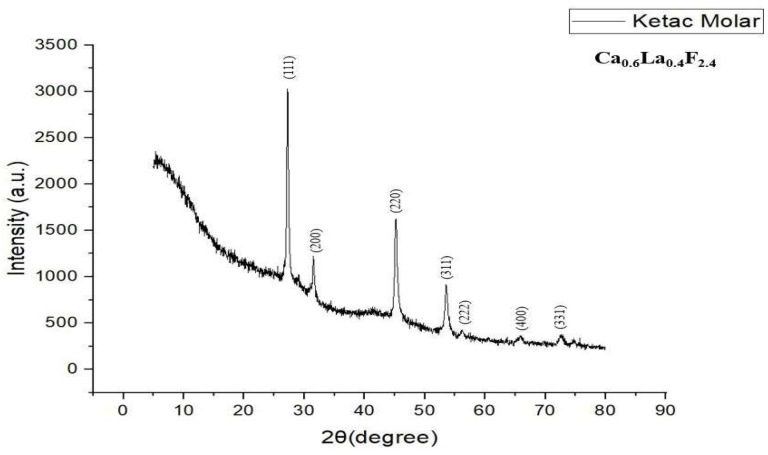
XRD analysis recorded for Ketac™ Molar EasyMix.

The X-ray diffractogram highlights a halo characteristic of vitreous phases (glasses) undergoing crystallization processes. The diffraction pattern of the thermally processed glass displayed prominent peaks at 2θ = 27.2°, 31.5°, 45.2°, 53.5°, 56.1°, 65.8° and 72.4°, corresponding to Miller indices (111), (200), (220), (311), (222), (400) and (331), indicating a complex combination containing calcium, fluoride and lanthanum with the chemical formula Ca_0.6_La_0.4_F_2.4_—calcium fluorolanthanate of cubic symmetry (Figure 5) [43].

Compared with the literature data and the American Society for Testing and Materials (ASTM 04-023-8279) standards [43], it is confirmed that Ketac^TM^ Molar EasyMix crystallizes in the cubic system. The sample exhibits a high crystallinity, evidenced by the high value of the maximum diffraction intensity, recorded at 3027, corresponding to the angle of 27.2°.

### 3.4. Metabolic Activity, Viability and Cytotoxicity Test Results

All test results were normalized to the control group, which was defined as 100% and consisted of cells not exposed to Ketac™ Molar EasyMix. The mean values for both control and experimental samples were calculated and expressed relative to the control group at each experimental level. This method was consistently applied across both MTT and NO assays. Statistical significance was assessed by direct comparison with the control group.

#### 3.4.1. MTT Assay

**Figure 6:**
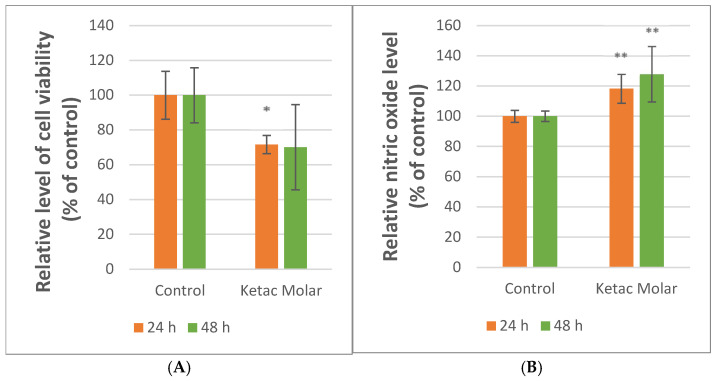
Cell metabolic activity and viability (**A**) and NO levels (**B**) were quantified after 24 and 48 h incubation in the presence of Ketac™ Molar EasyMix. Cells that have not been exposed to any dental material were used as the control group. The values are illustrated as mean ± SD results (MTT: *n* = 3; NO: *n* = 6). * *p* < 0.05 and ** *p* < 0.01 compared to control.

To assess the biological activity of the material, a quantitative MTT assay was conducted on human osteosarcoma cells to evaluate their mitochondrial metabolic activity and viability. According to Figure 6A, the MTT indicated that the cell’s metabolic activity decreased statistically significantly by ~28% for samples incubated with Ketac™ Molar EasyMix, compared to the control sample after 24 h (*p* = 0.029). After 48 h, a decrease of approximately 30% (*p* = 0.150) was observed (Figure 6A). According to the ISO 10993-5:2009 standard [44], materials that maintain cell metabolic activity and viability above 70% are regarded as biocompatible.

#### 3.4.2. NO Test

Regarding the Griess test, Ketac™ Molar EasyMix showed statistically significant increases in NO levels after the cells were incubated with the materials compared to the control sample for both 24 and 48 h (*p* = 0.002, *p* = 0.004). Supporting the cytotoxicity results, the elevated NO levels could serve as an indicator of cellular adaptation to stress associated with material exposure. (Figure 6B).

To enhance data integration and facilitate comparative analysis, the results have been organized in Table 2.

## 4. Discussion

Root perforations are iatrogenic or pathological lesions of the root wall that can compromise endodontic treatment outcomes and the integrity of periapical/periodontal tissues by enabling microbial leakage and sustaining local inflammation. Consequently, their clinical management depends on repair (and, when indicated, root-end) materials capable of providing effective sealing, biocompatibility, and long-term physicochemical stability under moisture- and blood-contaminated conditions typical of periodontal and surgical fields [6,7,8,28,30]. In this context, glass ionomer cements (GICs) continue to attract interest for selected endodontic defect-related applications because they can chemically adhere to mineralized tissues via ion-exchange mechanisms and can release ions (notably fluoride and calcium), which may modulate local biological responses in a formulation- and exposure-dependent manner [23,24].

An ideal material for perforation repair should limit bacterial leakage and establish a durable seal of the endodontic system while avoiding cytotoxic effects and excessive inflammation that might delay healing or lead to chronic issues. In biomaterial science, biocompatibility is commonly framed as “the ability of a material to perform with an appropriate host response in a specific application,” highlighting that biological acceptability is context-dependent (tissue type, clinical purpose, exposure time, and local environment) [45,46,47]. This framework is especially relevant for endodontic materials that come into direct contact with periapical tissues, where early inflammation could impair healing.

In the present study, we combined physicochemical characterization (SEM/EDX, FT-IR, XRD) with an in vitro biological screening using osteoblast-lineage-related cells (osteosarcoma cells—G292) to explore whether a conventional high-viscosity GIC (Ketac™ Molar EasyMix) exhibits a cellular response compatible with potential use in endodontic root defects. Because in vitro cytotoxicity outcomes can be influenced by cell type, assay endpoint, and experimental configuration, interpretation benefits from anchoring to standardized criteria such as ISO 10993-5:2009 [44].

In line with the study aim, the null hypothesis—that Ketac™ Molar EasyMix would not cause significant changes in cell viability or nitric oxide production compared to the control group—was disproven, as MTT values, although close to the ISO 10993-5:2009 threshold of 70%, were statistically lower than the control, while NO levels were significantly higher. These findings align with the material’s physicochemical characteristics, indicating that its surface properties and microstructure influence the cellular responses observed.

The exposure of G292 cells to Ketac™ Molar EasyMix resulted in a significant reduction in the MTT signal of approximately ~28% at 24 h (*p* = 0.029) and ~30% at 48 h compared with the control, while nitric oxide (NO) levels increased significantly at both time points (~18% at 24 h and ~28% at 48 h) (*p* = 0.002, *p* = 0.004). According to ISO 10993-5:2009, a reduction in viability of more than 30% (i.e., <70% of control) is considered cytotoxic under the applied test conditions [44]. Therefore, our MTT results place the material close to the 70% threshold, suggesting a borderline response in this specific direct-contact configuration.

This “near-threshold” pattern should be interpreted cautiously. ISO 10993-5:2009-based cytotoxicity testing is known to yield variable outcomes depending on extraction parameters, surface area-to-volume ratios, incubation conditions, and endpoint selection, and the standard does not fully eliminate inter-study comparability issues [44,48,49]. In this regard, the concomitant NO increase is an important complementary signal, as elevated NO in cell culture is commonly treated as an indicator associated with stress-related and inflammatory-associated cellular responses. Taken together, the MTT decrease and NO increase suggest that Ketac™ Molar exposure induces a measurable early cellular burden in osteoblast-lineage-related cells, even when metabolic activity remains near the ISO 10993-5:2009 acceptability threshold.

Recent studies on glass ionomer cements offer further context for understanding our in vitro results with Ketac™ Molar EasyMix. A 2024 study examined the cell viability of conventional GICs, including Ketac Molar EasyMix, in dental pulp stem cells. The researchers found reduced cell viability across all GIC formulations tested, including those enhanced with nanoparticles, implying that the material composition and ionic interactions affect cellular outcomes [50]. Conversely, the study conducted by Leenutaphong et al. demonstrated that Ketac™ Molar EasyMix maintained high cell viability, comparable to other materials in the same class during direct fibroblast assays, indicating that conventional GICs can sustain acceptable biological profiles under in vitro conditions [51].

Considering the variable findings reported in the literature, our results position Ketac™ Molar within an intermediate range of biological response. While some studies report more pronounced cytotoxicity and others indicate higher cell viability, our data showed viability values close to the ISO-defined threshold. Additionally, increased levels of nitric oxide were observed, which may suggest a cellular response to exposure to the materials.

In contrast, contemporary bioceramic materials, such as calcium silicate-based cements, including MTA and premixed putty calcium silicate cements, consistently demonstrate favorable biocompatibility and supportive cellular behavior in recent in vitro models. Additionally, the new premixed calcium silicate-based cements showed greater cell proliferation compared to MTA in dental pulp stem cells, without significant decreases in viability [52]. Systematic reviews of bioceramic responses further indicate that MTA and Biodentin support not only cell survival but also migration and differentiation in dental pulp stem cell cultures, emphasizing their high biocompatibility profile [53].

SEM images revealed a heterogeneous microstructure characterized by porosity and micro-roughness on both the surface and within the material. This is relevant from two complementary perspectives. First, surface chemistry and microtopography regulate protein adsorption and downstream cell signaling; in the general biomaterials literature, osteoblast adhesion, proliferation, and differentiation vary with micro-roughness and surface homogeneity, and moderately rough surfaces may reduce proliferation while promoting osteogenic differentiation markers [49,54,55]. Thus, the micro-rough, porous morphology observed here may increase the effective contact area and potentially support cell–material interactions.

Second, porosity and microcracking can facilitate fluid penetration and increase the effective surface area, potentially accelerating the diffusion of ions and other leachable species from a set/maturing cement into the surrounding environment. For conventional GICs, this may be particularly relevant in the early period after mixing, when the cement undergoes rapid and then continued maturation [56]. Consequently, a porous microstructure could amplify early exposure of adherent cells to transient chemical conditions (e.g., acidity and leachables), providing a plausible context for the observed MTT reduction and NO increase.

Conventional GICs set through an acid–base reaction between fluoroaluminosilicate glass particles and polyalkenoic acids, followed by maturation processes that continue after initial hardening and influence long-term properties and leachables [23,57,58]. In our study, the presence of characteristic FT-IR bands (including carboxylate-related vibrations) and bound water features is consistent with a mature/maturing ionomer network in which water participates in ongoing maturation and supports ion mobility [42,56].

In a porous material, local microenvironments may retain acidic components or facilitate their diffusion, potentially contributing to transient cellular stress. This mechanistic explanation is consistent with the known chemistry of conventional GICs and provides a conservative rationale for the early biological pattern observed (MTT reduction near threshold and increased NO) [59,60].

EDX indicated a high fluorine signal in the analyzed microarea (14.19 wt%), supporting the relevance of fluoride-related discussion. Fluoride release is a hallmark of GICs, but the magnitude and kinetics are strongly product-dependent and influenced by environmental conditions and maturation [23,42,56]. Importantly, product-specific comparisons have reported that Ketac™ Molar EasyMix exhibits among the highest fluoride release values within the tested GIC groups, with an increasing cumulative release over time [27]. These observations support the need to interpret early cellular responses in relation to potential fluoride exposure during prolonged contact.

At the cellular level, fluoride effects in bone-related models are dose- and context-dependent. While fluoride is beneficial in dental hard tissues, excess fluoride has been associated with adverse outcomes in osteoblast-related systems, including alterations of differentiation pathways and involvement of TGF-β1 signaling under certain experimental conditions [61]. In addition, in vitro cytotoxicity of conventional GICs varies across products and may be influenced by the profile of leachable components; studies on human gingival fibroblasts have documented material-dependent differences and discuss fluoride as one of the relevant factors among multiple possible contributors [34,62,63]. Our results might suggest that fluoride could contribute to the stress signature observed in our study conditions (MTT↓ and NO↑), through the direct contact of the cells with the fluoride-containing material surface and potential interactions at the cell–material interface.

EDX also detected lanthanum (13.56 wt% in the analyzed microarea), and XRD identified a crystalline phase consistent with calcium fluorolanthanate. These findings justify a biologically relevant discussion because lanthanum compounds have been described as concentration-dependent modulators of bone marrow stromal cell viability. Bai et al. reported increased viability at very low LaCl_3_ concentrations (10^−9^ M) and decreased viability with apoptosis at higher concentrations (10^−5^ M) under their experimental conditions [64]. Similarly, Lou et al. demonstrated improved proliferation and osteogenic differentiation in MC3T3-E1 cells for lanthanum-incorporated hydroxyapatite coatings when La-HA content remained below ~20% [65]. Broader analyses of lanthanide-substituted hydroxyapatite further support that lanthanide incorporation can modulate physicochemical properties and biological interactions, depending on composition and release conditions [65,66].

In the context of Ketac™ Molar, lanthanum-containing phases may thus represent a plausible factor influencing osteosarcoma-lineage responses, by affecting cells at the interface when in direct contact with the material surface.

A key strength of this work is the integrated approach that links microstructural and compositional analysis with early in vitro biological screening in osteoblast-lineage-related cells. At the same time, several limitations define the boundaries of interpretation. First, only one in vitro model (G292—osteosarcoma cells) was used; responses can differ across cell types (primary osteoblasts, BMSCs, fibroblasts, macrophage-lineage cells) and across endpoints beyond metabolic activity and NO release. Second, direct-contact exposure conditions can amplify early chemical effects; in vitro cytotoxicity outcomes are known to depend on the test setup, and ISO 10993-5:2009-based frameworks can yield non-identical outcomes across laboratories and configurations [44,67]. Third, the observed biological responses are also interpreted as resulting from direct contact between cells and materials, but the potential release of ions into the culture medium may also contribute. However, these ions were not quantified in the present study. Additionally, the present study is based on conventional 2D cell cultures, which do not fully replicate the complex three-dimensional microenvironment encountered by materials used in root perforation repair or retrograde fillings [68].

Accordingly, the present results should be viewed as a structured starting point sup-porting additional studies that: (i) quantify pH evolution and ion release (fluoride, calcium, aluminum, and lanthanum, where relevant) under identical surface area/volume and time conditions; (ii) incorporate extended osteogenic endpoints (ALP activity, mineralization assays, and osteogenic marker expression); (iii) include additional cell types and longer exposure windows relevant to periapical healing; (iv) compare direct-contact and extract-based approaches in alignment with ISO 10993-5:2009 testing logic; and (v) the use of in vitro tube models may provide a more physiologically relevant system for evaluating the biocompatibility of materials used in retrograde fillings and root canal perforations, allowing for a more accurate assessment of cellular responses to these materials [2,3,4,44,68,69].

## 5. Conclusions

In summary, Ketac™ Molar EasyMix exhibited a heterogeneous porous microstructure and a fluoride/lanthanum-containing composition, inducing a measurable early cellular stress signature in osteoblast-lineage-related cells (osteosarcoma cells), with significant MTT values close to the ISO 10993-5:2009 cytotoxicity threshold and a significantly increased NO release. These findings support further, product-specific research on Ketac™ Molar using experimental designs that more closely mimic the clinical conditions of perforation repair and surgical root-end environments.

## Figures and Tables

**Table 1:** The composition of the materials given by the manufacturer [36,37].

Material	Manufacturer	Composition	
Ketac™ Molar EasyMix	3M ESPE Germany	**Liquid**	**Powder**
		Water	Glass oxide (inert compounds)
		Copolymer of acrylic acid-maleic acid	
		Tartaric acid	

**Table 2:** The variation in the evaluated parameters expressed as % of the control (* *p* < 0.05, ** *p* < 0.001).

Incubation Time		24 h	48 h
Material/Test	Control	Ketac™ Molar EasyMix	
MTT	100%	71.64% *	70.10%
NO	100%	118.23% **	127.89% **

## Data Availability

The original contributions presented in the study are included in the article; further inquiries can be directed to the corresponding authors.

## Abbreviations

The following abbreviations are used in this manuscript:

MTA	Mineral trioxide aggregate
GICs	Glass ionomer cements
UV	Ultraviolet
SEM	Scanning electron microscopy
EDX	Energy dispersive X-ray spectrometer
FEG	Field-emission electron gun
FT-IR	Fourier-transform infrared spectroscopy
XRD	X-ray diffraction
G292	Human osteosarcoma cells
MTT	3-(4,5-dimethylthiazol-2-yl)-2,5-diphenyltetrazolium bromide
NO	Nitric oxide
SPSS	Statistical Package for the Social Sciences
ASTM	American Society for Testing and Materials
TGF-β1	Transforming growth factor-beta
MC3T3-E1	Subclone 4 preosteoblast cells
